# Toward adaptive radiotherapy for lung patients: feasibility study on deforming planning CT to CBCT to assess the impact of anatomical changes on dosimetry

**DOI:** 10.1088/1361-6560/aad1bb

**Published:** 2018-07-31

**Authors:** A J Cole, C Veiga, U Johnson, D D’Souza, N K Lalli, J R McClelland

**Affiliations:** 1 University College London Hospitals NHS Foundation Trust, 250 Euston Road, London, United Kingdom; 2 Centre for Medical Image Computing, Department of Medical Physics and Biomedical Engineering, University College London, Gower Street, London, United Kingdom; 3 St. Bartholomew’s Hospital, West Smithfield, London, United Kingdom; 4 Author to whom any correspondence should be addressed.; alison.cole2@bartshealth.nhs.uk

**Keywords:** deformable image registration, cone beam CT, adaptive radiotherapy, lung cancer

## Abstract

Changes in lung architecture during a course of radiotherapy can alter the planned dose distribution to the extent that it becomes clinically unacceptable. This study aims to validate a quantitative method of determining whether a replan is required during the course of conformal radiotherapy. The proposed method uses deformable image registration (DIR) to flexibly map planning CT (pCT) data to the anatomy of online CBCT images. The resulting deformed CT (dCT) images are used as a basis for assessing the effect of anatomical change on dose distributions. The study used retrospective data from a sample of seven replanned lung patients. The settings of an in-house, open-source DIR algorithm were first optimised for CT-to-CBCT registrations of the anatomy of the thorax. Using these optimised parameters, each patient’s pCT was deformed to the CBCT acquired immediately before the replan. Registration accuracy was rigorously validated both geometrically and dosimetrically to confirm that the dCTs could reliably be used to inform replan decisions. A retrospective evaluation of the changes in dose delivered over time was then carried out for a single patient to demonstrate the clinical application of the proposed method. The geometric analysis showed good agreement between deformed structures and those same structures manually outlined on the CBCT images. Results were consistently better than those achieved with rigid-only registration. In the dosimetric analysis, dose distributions derived from the dCTs were found to match closely to the ‘gold standard’ replan CT (rCT) distributions across dose volume histogram and absolute dose difference measures. The retrospective analysis of serial CBCTs of a single patient produced reliable quantitative assessment of the dose delivery. Had the proposed method been available at the time of treatment, it would have enabled a more objective replan decision. DIR is a valuable clinical tool for dose recalculation in adaptive radiotherapy protocols for lung cancer patients.

## Introduction

Radiotherapy treatment planning is performed on a ‘snapshot’ of the patient anatomy before treatment commences, the planning CT (pCT). The approved plan is then delivered via multiple fractions over the course of several weeks. However, progressive anatomical changes may occur during treatment which cause the delivered dose distribution to differ from the planned dose distribution. This is a particular issue for lung patients in whom non-negligible tumour positional changes and size reductions are often observed (McDermott *et al* 2006, Britton *et al* 2007, Sonke *et al* 2008, Kwint *et al* 2014). The problem is compounded by the fact that adjacent tissues in the thorax often have very different electron densities, which means even small structural differences could have a non-negligible impact on dose.

These day-to-day variations in anatomy have driven the development of adaptive radiotherapy (ART), where a new plan (the replan) is introduced at a time during treatment when the dose distribution delivered by the original plan has become clinically unacceptable. Unfortunately it can be difficult to identify the most appropriate time to intervene, as changes in doses to target structures and organs at risk (OARs) can only be quantified by comparing the original and current dose distributions. CT imaging is the gold-standard method to quantify electron density and hence generate accurate dose distributions. However, repeat CT imaging is burdensome in a busy clinical department, and would increase the patient’s concomitant dose. In recent years, 3D imaging data from on-treatment cone-beam CT (CBCT) systems has become increasingly available. This modality was initially introduced to improve image guidance for patient setup, but has proven to be a useful tool in monitoring anatomical change over the course of treatment. There are several advantages to using CBCT over repeat CT data for ART—it represents the patient anatomy in treatment position, there is no need for additional imaging appointments, and the concomitant dose is reduced. Unfortunately, increased scatter obstructs the signal, leading to lower image quality than for standard CT (Siewerdsen and Jaffray 2001, Scarfe and Farman 2008). This means the derivation of electron density distributions and dose information from CBCT hounsfield unit (HU) data using CT-based methods can lead to inaccuracies, particularly in regions of high tissue inhomogeneity (Ma *et al* 2014a). Various authors are working on correction strategies that would minimise these inaccuracies (Yoo and Yin 2006, Marchant *et al* 2008, Richter *et al* 2008, Poludniowski *et al* 2009, Xu *et al* 2015, Kurz *et al* 2016, Thing *et al* 2017, Joshi *et al* 2017). However, this has proved challenging in regions of tissue inhomogeneity such as the thorax. Additionally, these methods do not address the time-consuming and difficult contouring on the noisy CBCT image.

An alternative approach uses CBCT data indirectly as an anatomical template to which the electron density data derived from the pCT is deformably registered. This prevents CBCT artifacts from influencing the calculated dose distributions and has the additional benefit of automatically propagating the previously outlined structures. CBCT is affected by shading, clipping and radar artifacts in addition to scatter. DIR-based approaches have been implemented in several recent ART studies; to produce a ‘ground truth’ against which CBCT-based dose calculations can be validated (Yang *et al* 2007), to propagate contours from CT to CBCT data in order to re-evaluate OAR doses (Ma *et al* 2014b), as a basis for CBCT scatter corrections (Kurz *et al* 2016), to validate particular DIR algorithms for other sites (Moteabbed *et al* 2015, Veiga *et al* 2014) and as part of adaptive stereotactic and proton workflows (Han *et al* 2015, Zhang *et al* 2015, Veiga *et al* 2016). However, until now, CT–to-CBCT DIR validation on real lung patient data has only been performed in the context of passively scattered proton therapy (Veiga *et al* 2017), a treatment modality with stricter patient inclusion criteria with regards to breathing motion. Therefore, the accuracy and robustness of this approach when applied to lung patients treated with conformal radiotherapy has not yet been established.

In this study, we validate and demonstrate a DIR-based ART workflow tailored for conformally treated lung cancer patients. To ensure the reliability of the method, rigorous validation of the accuracy of the registrations has been carried out, first geometrically by assessing structure deformations, and then dosimetrically through comparisons with gold standard replan data. The feasibility of using deformed structures rather than directly outlined structures to produce dose volume histogram (DVH) information has also been tested. To conclude the study, the clinical application of the proposed method has been demonstrated via a weekly retrospective assessment of the influence of anatomical change on the planned dose distribution for a single patient.

## Materials and methods

### Patient data

Seven lung cancer patients treated with conformal external beam radiotherapy were selected, all of whom were re-planned during the course of treatment due to anatomical changes (table 1). The imaging protocol for these patients consisted of CT images acquired at planning (pCT) and adaptive stages (replan CT, rCT) and weekly cone-beam CTs (CBCTs) for image-guidance and offline review purposes. Under existing protocols, the decision to replan was made by a clinician based on a visual assessment of the degree of anatomical change.

**Table 1.** Patient summary. Examples of anatomical charge are provided at figure 1.

Patient number	M/F	Age at pCT scan	CTV volume on pCT (cm^3^)	Original plan treated fractions	Replan treated fractions	Reasons for replan
1	M	53	33.7	6	26	Partial lung reinflation following atelectasis. Moderate anatomical change.
2	F	63	92.3	14	18	Increased tumour volume within region of atelectasis. Minor anatomical change.
3	M	46	659.9	15	17	Reduction in tumour size. Major anatomical change.
4	M	68	100.5	15	17	Tumour positional change. Minor anatomical change.
5	M	75	235.0	24	8	Tumour size reduction superiorly. Moderate anatomical change.
6	M	77	358.2	9	19	Superior and anterior changes in tumour volume. Moderate anatomical change.
7	F	81	87.4	9	11	Tumour positional change, posterior migration. Minor anatomical change.

**Figure 1.**
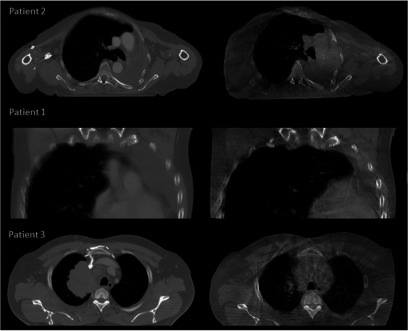
Examples of minor (Patient 2), moderate (Patient 1) and major (Patient 3) anatomical changes. For each patient the pCT is shown on the left and the CBCT is shown on the right.

The CT images were acquired on a GE Lightspeed Widebore 16 slice CT system (GE Healthcare, Buckinghamshire, UK). For one of the seven patients, the pCT and rCT were helical images and for the remaining six they were 4DCT Average Intensity Projection (AvIP) images. In all cases the pCT and rCT were the images on which dose calculations were originally performed. CBCT images were acquired using the Varian OBI version 1.4 (Varian Medical Systems, Palo Alto) in full rotation half-fan mode with the following parameters; 45 cm FOV, 110 kV, 20 mA and 20 ms. The CBCT acquired immediately prior to the replan was selected for analysis; the time delay between acquisition of the CBCT and rCT varied between zero and five days.

### NiftyReg optimisation

NiftyReg^4^4http://cmictig.cs.ucl.ac.uk/research/software/software-nifty/niftyreg (Modat *et al* 2010) is an open source DIR algorithm that uses the well-established free-form deformation technique (Rueckert *et al* 1999) in which B-spline functions are used to parameterise a mapping between the images. The algorithm has been independently validated on thoracic data as part of the EMPIRE10 challenge (Murphy *et al* 2011). The registration pipeline starts with a rigid registration between CBCT and pCT (Ourselin *et al* 2001), followed by deforming the pCT to the CBCT.

The same set of DIR parameters were applied across the range of patients; these were extensively optimised on a sub-group of the cohort in preliminary experiments. The velocity field parameterisation of the DIR algorithm was used which guarantees that the transformations are symmetric, inverse-consistent, and diffeomorphic (Modat *et al* 2011). The local normalised cross correlation similarity measure was used, as it assumes a local linear relationship between the image intensities, but can account for different regions of the images having different intensities for the same tissue type (as is often the case for CBCT images) (Cachier *et al* 2003). A multi-resolution approach with three levels and a fine grid spacing of eight voxels was employed and the registrations were regularised via the bending energy penalty term with a relative weight of 1%. To enable the algorithm to distinguish between the real edge of the patient and the CBCT field of view (FOV) edge, voxels outside the FOV were masked and ignored during the registration.

### Geometric validation

**Figure 2.**
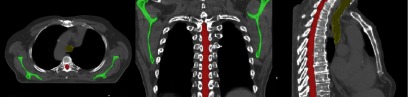
Scapulae (green), spinal canal (red) and trachea (yellow) as outlined on the pCT for a single patient.

The geometric validation is an initial test of the ability of the registration tool to map matching anatomical features between the images. To validate the registrations geometrically, three sample structures—the scapulae, spinal canal and trachea—were directly outlined on both the pCTs and CBCTs for each patient in accordance with clinical protocols. These particular structures were chosen because they were easily identifiable on the CBCT images (figure 2).

The pCT structure sets were then deformed by applying the transformation obtained from the optimised registrations. Deformed structures were cropped to match the CBCT structures in length where necessary.

For each patient, three comparison metrics were then calculated to assess how well the deformed pCT structures matched the directly outlined gold standard CBCT structures. The metrics are defined as follows (where $A$ and $B$ are the sets of voxels that define the volumes, $\tilde{A}$ and $\tilde{B}$ are the corresponding surfaces, ${{a}_{C}}$ and ${{b}_{C}}$ are the centroid positions of sets $A$ and $B$ respectively), as in Veiga *et al* (2014):

1. $ {\rm Dice}\,{\rm Similarity}\,{\rm Coef\,ficient}\!: $ $ {\rm DSC}=\frac{2\left| A\cap B \right|}{\left| A \right|+\left| B \right|}$
2. ${\rm Distance}\,{\rm Transform}~\left( {\rm a}.{\rm k}.{\rm a}.\,{\rm Distance}\,{\rm To}\,{\rm Agreement} \right)\!:$ $DT\left(a \right)=min\left(\Vert a-b \right\vert\Vert),~a\in \tilde{A},~b\in \tilde{B}$
3. ${\rm Centroid}\,{\rm Position}\,{\rm Error}\!:$${\rm CPE}=\Vert{{a}_{C}}-{{b}_{C}}\Vert.$

For comparison purposes, the pCT was also rigidly aligned to the CBCT and the associated structures were compared to the gold standard using the same metrics.

### Dosimetric validation—voxelwise dose differences

The dosimetric validation tests the reliability of dose calculations carried out on the registered image data. To validate the registrations dosimetrically, dose distributions calculated on the dCT images were compared with gold standard dose distributions calculated on the rCTs. To obtain the gold standard distribution, rCT images were rigidly registered to pCT images manually by a single operator. Registrations matched the bony anatomy in the region of the tumour in line with clinical protocols, the aim being to create a gold standard dose distribution that reflected anatomical changes only, not setup errors.

**Figure 3.**
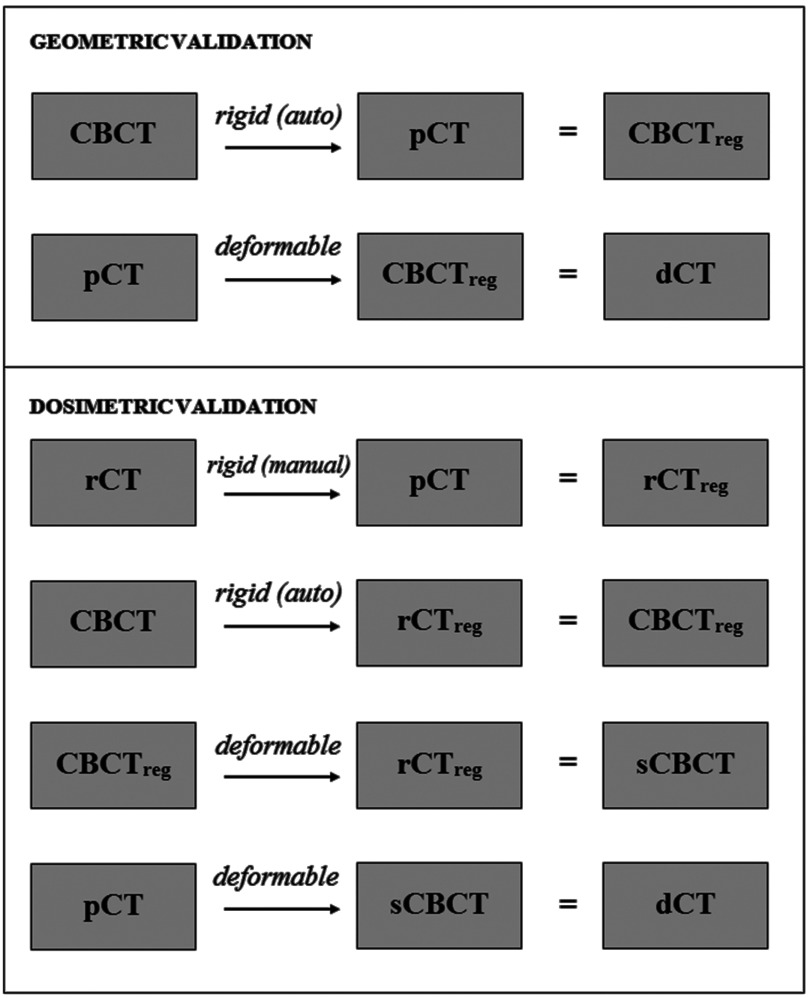
Pipeline of the registrations carried out to produce deformed pCT images (dCTs) for validation.

Each CBCT-rCT pair was acquired using different modalities at different times, such that non-negligible positional and anatomical differences existed between these images. This meant that even if the pCTs were perfectly deformed to the CBCTs, the resulting dCT images would still differ from the gold standard rCT images. To mitigate the corrupting effects of these differences on the dosimetric validation, simulated CBCT (sCBCT) images were first produced by deformably registering the CBCTs to the rCTs (Veiga *et al* 2014). These sCBCTs were then used in place of the original CBCT images as the targets for the deformation. All sCBCTs were visually inspected to ensure they represented the rCT anatomy more closely than the original CBCT images. The dCT images were then created by registering the pCTs to the sCBCTs. A summary of the registrations that were carried out to produce the deformed images is shown at figure 3.

Dose calculations were carried out on the Eclipse (Varian Medical Systems, Palo Alto, CA) treatment planning system (TPS) using the analytical anisotropic algorithm. Details of the original pCT treatment plan, including beam arrangement, fluence maps and monitor units were copied onto the dCT and rCT. All dose calculations were performed at a resolution of 1 mm.

The rCT and dCT dose distributions were then exported and a voxelwise dose difference was computed over two regions defined on the rCT as follows:

1. The region exceeding 95% of the prescribed dose (the Treated Volume, TV).
2. The region exceeding 50% but less than 95% of the prescribed dose (the High Dose Region Volume, HDRV).

A potential issue for any method that uses CBCT data as a basis for dose calculations is the restricted FOV. Treatment fields cannot pass through regions of missing data, so this must be obtained from elsewhere. In this study, the anatomy outside the CBCT FOV was estimated by applying a smooth transition between deformed pCT slices within the FOV and rigidly aligned pCT slices outside it (Veiga *et al* 2014).

### Dosimetric validation—DVH data

To further assess the similarity of the dCT and rCT dose distributions, a comparison of DVH data derived from those distributions was carried out. Four clinical structures that had been outlined on the rCT at the time of treatment were used as a basis for the DVHs—the heart, lungs (combined lung volume minus GTV, excluding all regions of atelectasis in accordance with clinical protocols), spinal canal  +  5 mm (spinal canal isotropically grown by 5 mm) and CTV  +  1 cm (CTV isotropically grown by 1 cm). To prevent geometric uncertainties from confounding the comparison, the exact same structures were used to derive DVHs from both the dCT and rCT dose distributions.

A further comparison was also carried out to investigate the feasibility of using deformed pCT structures as the basis for dCT DVHs as, in a real clinical scenario, no rCT structures would be available at the time the dose distribution was being evaluated. To do this, the deformation matrix was applied to the relevant clinical pCT structures (heart, lungs, spinal canal and CTV). The same geometric margins were then applied to the deformed spinal canal and CTV to produce a set of structures that were analogous to the clinical rCT structures. As the volumetric tolerances (V20 and V40, defined as the percentage volume receiving a dose of 20 and 40 Gy or more respectively) depend on the total volume of the structure, the heart and lung calculations were adjusted to remove the effect of differences in volume attributable to the smoothed region outside the sCBCT FOV.

### Demonstration of clinical application

**Figure 4.**
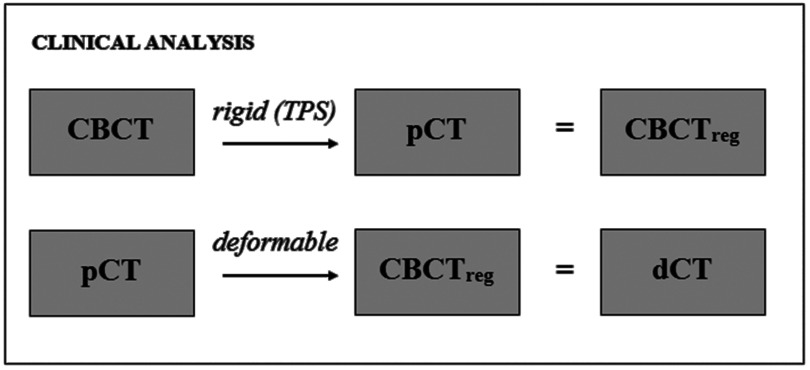
Pipeline of the registrations carried out to produce deformed pCT images for clinical analysis.

Following validation of the accuracy of the registrations, the intended clinical procedure was demonstrated retrospectively for a single patient within the sample (Patient 5). Four CBCTs that were obtained prior to the patient replan (including the one used in the validation exercises above) were used to generate dCTs. For this particular analysis, the CBCT was first rigidly registered to the pCT using online registration parameters obtained from the TPS (figure 4). This ensured that the dose distribution derived from the dCT also incorporated residual setup errors, as it would in a real clinical scenario. Dose distributions and DVHs were calculated using deformed pCT structures. A visual appraisal of the deformed structures was carried out with reference to both the dCT and CBCT to check for any obvious mismatches in anatomy.

Changes to the dose distribution over the course of treatment were then evaluated, taking into consideration the clinical objectives for this patient’s treatment plan.

## Results

### Geometric validation

**Figure 5.**
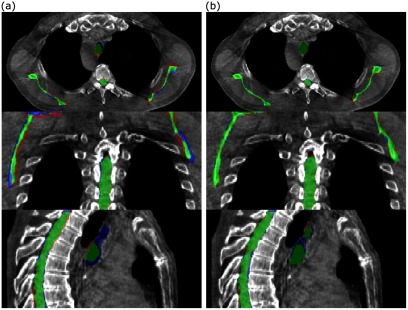
Comparison of (a) rigidly aligned and (b) deformed structures with the directly outlined CBCT gold standard for a single patient. Green represents areas covered by both sets of structures, blue represents areas covered by the directly outlined structures only and red represents areas covered by the rigidly aligned/deformed structures only.

Visual assessment suggested that the structure sets deformed from the pCTs matched well with the gold standard structures directly outlined on the CBCT (figure 5).

The mean results across the seven patient sample for the three volumetrically outlined structures are summarised in table 2.

**Table 2.** Geometric validation results; DT  >  2 mm  =  the percentage of DT values that exceeded 2 mm. |DT| 95%  =  the 95% percentile of the absolute DT values. |DT| mean  =  the mean absolute DT value. |DT| s.d.  =  the standard deviation of the absolute DT values. All statistics were calculated per patient. The table shows the mean  ±  standard deviation of each statistic over the seven patients.

	Scapulae		Spinal canal		Trachea		All Structures	
	Rigid	Deformed	Rigid	Deformed	Rigid	Deformed	Rigid	Deformed
DSC	0.73 ± 0.07	0.883 ± 0.011	0.85 ± 0.03	0.898 ± 0.014	0.71 ± 0.14	0.88 ± 0.03	0.77 ± 0.11	0.889 ± 0.021
CPE (mm)	5 ± 3	3.0 ± 1.4	2.0 ± 1.9	1.6 ± 1.0	5 ± 3	1.4 ± 0.7	4 ± 3	2.0 ± 1.3
DT > 2 mm (%)	20 ± 11	2.6 ± 2.0	7 ± 5	2.2 ± 1.4	41 ± 17	13 ± 10	21 ± 18	5 ± 7
|DT| 95% (mm)	3.9 ± 1.2	1.8 ± 0.3	2.4 ± 0.5	1.8 ± 0.4	7 ± 3	3.2 ± 1.0	4 ± 3	2.2 ± 0.9
|DT| mean (mm)	1.4 ± 0.4	0.59 ± 0.07	0.91 ± 0.19	0.61 ± 0.07	2.5 ± 1.1	1.1 ± 0.4	1.5 ± 0.9	0.7 ± 0.3
|DT| s.d. (mm)	1.3 ± 0.4	0.73 ± 0.12	0.81 ± 0.14	0.64 ± 0.08	2.4 ± 0.9	1.3 ± 0.6	1.4 ± 0.8	0.8 ± 0.4

The deformed structures performed consistently better than the rigidly aligned structures for each of the metrics measured. For every structure, a DSC of 0.88 or greater was achieved by the deformable registration. The results for the spinal canal were better than for the other structures, which is unsurprising given the relative rigidity of the structure, its insensitivity to setup differences and its clarity on CBCT images. The trachea produced the most variable results across patients, as evidenced by the relatively high standard deviations for each of the metrics. This soft tissue structure underwent the most anatomical change across the patient dataset and so presented the greatest challenge for the registrations. The scapulae Could you use plural scapulae throughout to be consistent produced well-matched results across the patients for deformed structures which were comparable to those for the spinal canal. During our preliminary DIR parameter optimisation work we found the scapulae, on visual appraisal, to be particularly sensitive to registration errors. This was also found in a previous study by Veiga *et al* (2016).

### Dosimetric validation—voxelwise dose differences

**Figure 6.**
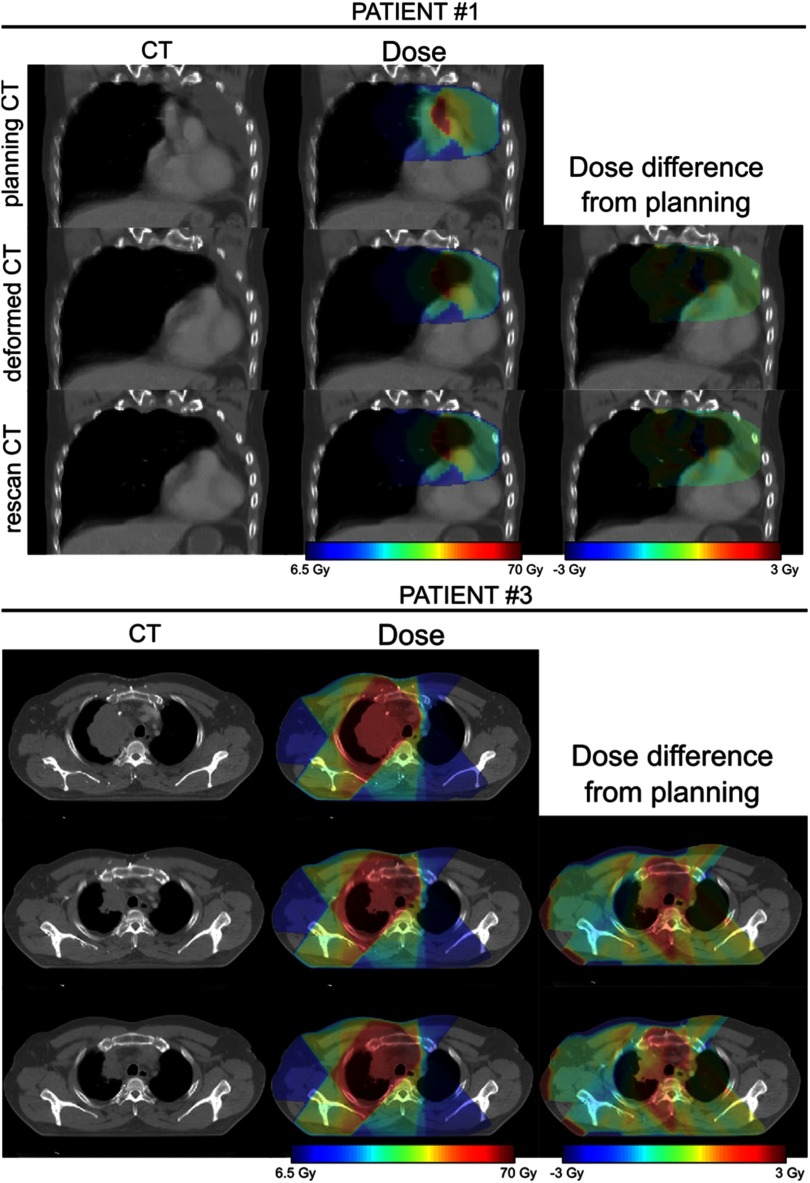
Comparison of rCT and dCT CT scans and dose distributions (against pCT) for two of the patients included in the study. In each case the prescription dose was 64 Gy.

The dCT images were well-matched to the rCT images and the associated dose distributions for each patient were visually indistinguishable from the rCT gold standards (figure 6).

**Figure 7.**
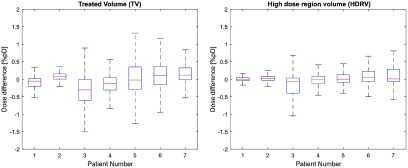
Boxplots showing the distribution of voxelwise dose differences for each patient for the TV (treated volume—region exceeding 95% of the prescribed dose) and the HDRV (high dose region volume—region exceeding 50% but less than 95% of the prescribed dose). Outliers corresponding to points outside the  ±2.7 s.d. range have been suppressed.

The results of the voxelwise dose difference analysis for the two selected regions are presented at figure 7.

### Dosimetric validation—DVH data

A summary of deviations of relevant dCT DVH values from the rCT gold standard DVH values across patients is included at table 3.

**Table 3.** Summary of absolute percentage deviations of dCT DVH values from the rCT gold standard values. dCT DVHs were calculated using directly outlined rCT structures (columns 1–3) and deformed pCT structures (columns 4–6). Maximum dose values are expressed as a normalised percentage of the prescribed dose. All values are given to one decimal place.

	rCT STRUCTURES			DEFORMED STRUCTURES		
	Mean deviation %	S.D. deviation %	Max deviation %	Mean deviation %	S.D. deviation %	Max deviation %
CTV + 1 cm max	0.3	0.2	0.7	0.7	0.7	1.6
CTV + 1 cm V95%^a^	0.7	0.7	2.0	12.0	8.2	25.8

Spinal canal + 5 mm max	0.3	0.3	0.7	2.2	3.3	9.4

Lung^b^ max	0.3	0.2	0.7	0.5	0.4	1.2
Lung^b^ V20	0.1	0.1	0.3	0.8	1.2	3.0

Heart max	0.3	0.2	0.6	1.0	0.8	2.3
Heart V40	0.0	0.0	0.1	2.0	2.9	8.5

a V95%  =  percentage of structure covered by at least 95% of the prescribed dose.

b Lung combined minus GTV. Regions of atelectasis excluded from volume (in accordance with clinical protocols).

For DVHs calculated using the rCT structures, deviations from the gold standard rCT values were small across all patients. The largest deviation of 2.0% related to the CTV  +  1 cm V95% for a single patient where there was a major reduction in tumour size in the CBCT which was not fully captured in the dCT. For this patient the need for a replan would be clear from visual inspection of the images.

**Figure 8.**
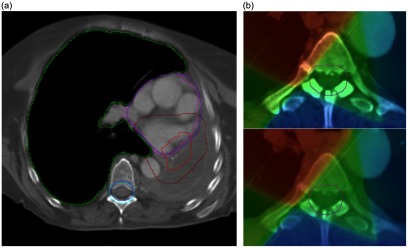
(a) Deformed pCT (light) and directly outlined rCT (dark) structure comparison for a single patient—the right lung (green), spinal canal  +  5 mm (blue), heart (purple) and CTV  +  1 cm (red) are overlaid for both structure sets on a single transverse slice. Whilst the lung and spinal canal are similar, the CTV  +  1 cm outlined on the rCT is much larger than the deformed CTV  +  1 cm. Although the heart appears well-matched on this slice, the heart that was outlined on the rCT extends further superiorly than the deformed structure. (b) Directly outlined spinal canal  +  5 mm (top) compared with deformed spinal canal  +  5 mm (bottom). Although the structures are similar, there is a noticeable difference in maximum dose as the directly outlined rCT structure slightly overlaps the treatment field whereas the deformed structure does not.

The DVH values that were calculated using the deformed pCT structures were clearly less well matched to the gold standard. On visual inspection, it was clear that the deformed structures were, in some cases, very different to the directly outlined rCT structures (figure 8(a)). The reasons for this are discussed in detail in the following sections. In addition, for a single patient, there was a large spinal canal  +  5 mm maximum dose deviation of 9.4% which occurred even though the structures were visually very similar. Here, the directly outlined structure slightly overlapped into a treatment field whereas the deformed structure did not (figure 8(b)). The implications of this are also considered in the next section.

**Figure 9.**
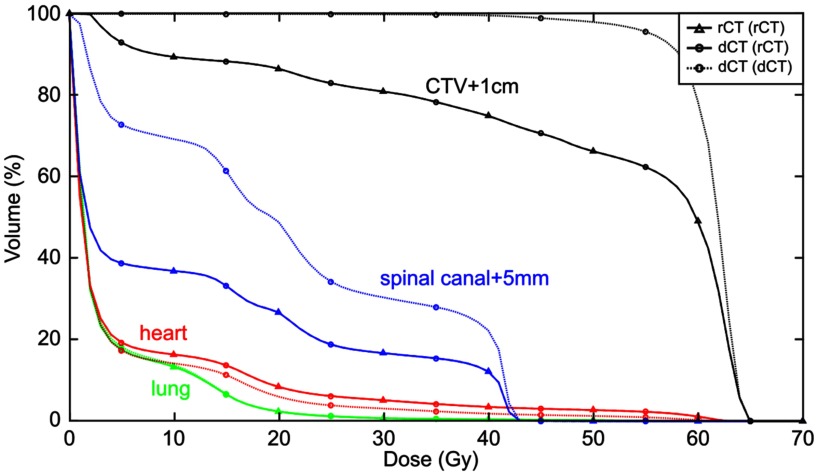
Gold standard rCT DVH overlaid with dCT DVHs calculated using either directly outlined rCT structures ‘dCT (rCT)’ or deformed pCT structures ‘dCT (dCT)’ for a single patient. The dCT (rCT) matches the rCT DVH well, whereas the dCT (dCT) is noticeably different for all structures with the exception of the lungs. It should be noted that spinal canal  +  5 mm differences are largely attributable to outlining differences—the rCT structure extends further than the pCT (and hence the deformed) structure in both the superior and inferior directions. However, this is not an issue as only Dmax is considered for serial structures such as this.

A comparison of the rCT gold standard DVH with dCT DVHs using both directly outlined rCT structures and deformed pCT structures is shown at figure 9.

### Demonstration of clinical application

To demonstrate the clinical application of the DIR method, we performed serial dose calculations on CBCT anatomy on Patient 5, a subject that appeared to demonstrate a steady decrease in tumour size over the course of four weeks. As there were no obvious sudden changes in tumour size from one week to the next it was not clear from visual assessment at what time point a replan might be appropriate.

The effect of anatomical change on the dose distribution as suggested by our proposed method is illustrated in table 4 below. Care must be taken in the interpretation of these results, particularly in light of our findings above regarding uncertainties surrounding deformed target structures and those located in homogenous regions. In a real clinical scenario we would strongly recommend that the clinician carries out a review of all deformed structures and outlines a new CTV (and OARs where relevant) for the purposes of dosimetric evaluation, using information from the original and deformed GTV and CTV structures along with visual information from the CBCT and dCT images. However, in the absence of such a review having been undertaken for our analysis, we have included DVH statistics derived from both the originally outlined pCT CTV and the deformed CTV. While this would not be relied upon clinically, the data does serve to illustrate the type of information that may be derived under our proposed method.

**Table 4.** Changes in relevant DVH statistics over the course of treatment calculated from dCT images for a single patient. All values are given to one decimal place.

	pCT		dCT Week 1		dCT Week 2		dCT Week 3		dCT Week 4	
	%	Gy	%	Gy	%	Gy	%	Gy	%	Gy
CTV (original) max	107.2	68.6	107.1	68.5	106.7	68.3	107.1	68.6	108.4	69.4
CTV (original) V99%^a^	96.2	—	92.7	—	92.3	—	96.3	—	96.1	—
CTV (deformed) max	107.2	68.6	107.0	68.5	106.5	68.2	107.1	68.6	107.9	69.0
CTV (deformed) V99%^a^	96.2	—	94.8	—	94.5	—	95.0	—	96.1	—
Spinal canal max	71.6	45.8	71.7	45.9	72.1	46.1	71.4	45.7	71.0	45.4
Spinal canal + 5 mm max	74.5	47.6	75.0	48.0	89.2	57.1	91.9	58.8	73.3	46.9
Lung^b^ max	106.0	67.9	105.7	67.7	105.0	67.2	105.4	67.5	106.5	68.2
Lung^b^ V20	19.5	—	18.1	—	17.8	—	19.2	—	18.1	—
Heart max	106.4	68.1	106.3	68.1	105.4	67.5	105.5	67.5	107.0	68.5
Heart V40	7.1	—	7.0	—	8.7	—	9.8	—	7.7	—

a V99%  =  percentage of structure covered by at least 99% of the prescribed dose.

b Lung combined minus GTV. Regions of atelectasis excluded from volume (in accordance with clinical protocols).

**Figure 10.**
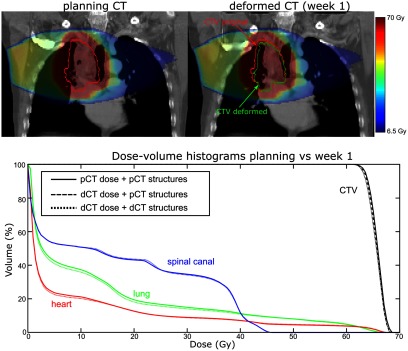
(Top) dose distributions calculated on the pCT and week 1 dCT and (bottom) dCT DVHs overlaid with the pCT DVHs.

The CTV data suggests there was a reduction in target coverage early on in the treatment with V99% for the original CTV structure dropping from 96.2% to 92.7% in Week 1. Dose distributions as calculated on the pCT and the Week 1 dCT are shown at figure 10.

A reduction in GTV size is evident on visual inspection of the two images. As a result, the proportion of soft tissue present within the original CTV volume was less on the Week 1 dCT image than on the pCT image. As soft tissue absorbs dose to a greater extent than lung tissue, V99% coverage of the original CTV structure was correspondingly reduced. Unlike the original CTV structure, the deformed CTV reflected the reduction in GTV size, which meant that V99% coverage for this structure only reduced to 94.8%.

While these results demonstrate the type of quantitative information that may be derived from our method, it is important to note that neither the original, nor deformed, CTV is representative of the actual clinical CTV at that point in time and they should not be relied on as such. What these structures can do, however, is provide information to the clinician to assist in the outlining of an appropriate CTV for dose evaluation. This is discussed further in the section below.

Due to the proximity of the target volume to the spinal canal there was a clear risk that anatomical change would cause this volume to breach the clinical tolerance (47 Gy  <  0.1 cc). Table 4 includes data for both the spinal canal and the spinal canal PRV structure (spinal canal  +  5 mm) which suggests that, while the PRV structure breached tolerance in both Week 2 (*D*_max_  =  57.1 Gy) and Week 3 (*D*_max_  =  58.8 Gy), the spinal canal volume itself remained well within tolerance. This information would prove useful to the clinician in deciding if a replan is necessary. Other OAR structures remained well below tolerance (Lung V20  <  30%, Heart V40  <  30%).

## Discussion

The results of this study suggest that there is a role for DIR algorithms in adaptive photon radiotherapy processes for lung cancer patients. For each of the patients in the sample tested, the geometric and dosimetric data derived from the dCT images was found to be sufficiently accurate to inform a replan decision, provided proper consideration is given to the structures used for dosimetric evaluation. Further, the results indicate that universal image registration parameters, as long as tailored for the data generated at each centre, are likely to be suitable across a range of patients. This finding should greatly reduce the resource requirements for clinical implementation. Given the length of time taken to produce accurate registrations (approximately 30–300 min depending on the processing power) online review is not yet feasible. However, it is expected that calculation speed will be increased in the future to make this a possibility.

In validating the algorithm, several (but not all) of the recommendations of the AAPM TG-132 (Brock *et al* 2017) have been followed. The guidance states that ‘the scope of the verification tests will depend on .. the clinical purpose of image registration’ (Brock *et al* 2017, p53). In this case, the proposed clinical purpose does not require the voxel-wise correspondences to be 100% ‘correct’, so long as the information derived from it can be used to assist the clinician’s decision as to whether the patient should have a new CT. Indeed, when registering images where anatomical changes have occurred, the ‘correct’ voxel-wise mapping is not well-defined, as there will be tissue in one or both images that is not present in the other. We have therefore only used validation metrics that we consider to be meaningful in the context of our proposed workflow.

The majority of studies involving DIR methods for lung patients have not extensively tested the reliability of information derived from deformed data in this way. Yang *et al* (2007) used deformed image data as the ‘ground truth’ in a study that looked to validate CBCT-based dose calculations for three prostate patients and a single lung patient. However, the accuracy of the deformation algorithm was not validated specifically for CT-to-CBCT lung patient data. Zhang *et al* (2015) tested the feasibility of using a novel motion modelling and deformation technique for online verification of stereotactic lung doses. This technique did involve deformation of CT to CBCT data and was tested by way of both digital and physical phantom studies. However, no validation was carried out on real lung patient data. Ma *et al* (2014b) carried out a study in which OAR doses were retrospectively adapted by re-optimising treatment plans using pCT contours that had been deformed to the most recent CBCT online data. Accumulated doses were then compared to those obtained by applying the original treatment plans to the deformed contours. While this study demonstrates a potential application for DIR in adaptive planning, it lacks validation of the accuracy or reliability of the registrations. Additionally, changes in the distribution of tissues of different densities were not accounted for in the dose calculations as these were carried out on the original pCT images. Han *et al*’s (2015) study of SABR treatments used a different approach, whereby the impact of changing anatomy on planned dose distributions was retrospectively assessed by dose calculations carried out directly on the deformed pCT images. Here, dosimetric uncertainty in the calculations was quantified using a rigid anthropomorphic phantom to assess differences in the mean HU values between original and deformed data. However, as the phantom CT and CBCT scans were anatomically identical, the errors and uncertainties from the phantom study may not give a good representation of the errors that occur when using patient data in which anatomical differences are present—in order to confirm this, validation is necessary on the patient data itself. Rigorous validation of deformations on real lung patient data has been carried out for a cohort of patients who were treated with passive scatter proton therapy (Veiga *et al* 2017). However, our separate validation study was necessary to demonstrate that DIR can be similarly effective in the context of lung photon treatments for two reasons: (1) photon and proton dose distributions are intrinsically different in terms of robustness to anatomical change, and (2) the proton patient cohort in Veiga’s study represented a restrictive subset of the wider photon patient cohort (i.e. only small tumours with a small amplitude of movement were treated).

There are certain limitations to our study that must be considered. Although the deformations proved successful for a sample of seven patients using a well-defined set of registration parameters, it does not follow that these parameters can be applied for all patients. The accuracy achieved will vary with the quality of the CBCT and the degree of anatomical change. For example, large scale anatomical changes such as those that occurred in Patient 3 present a greater challenge for the DIR. In certain cases, this may influence the results to the extent that the dCTs can no longer produce reliable dosimetric information. For this reason, registration results must always be subjected to some form of appraisal before they are used, in order to identify situations where the algorithm has underperformed. Generally this would be done by way of visual appraisal by a trained operator, although automated methods for aiding the detection of registration errors are in development (Paganelli *et al* 2013, Beasley *et al* 2016). However, we expect that for severe cases where the parameters cannot be adjusted to produce an acceptable result, a replan will almost certainly be required based on visual inspection.

Our study deforms AvIP data to 3D CBCT data and performs dose calculations on the resulting deformed AvIP data. In both of these modalities, breathing motion can lead to blurring of the images, and this could introduce inaccuracies to registrations and/or dose calculations. However, visual inspection of the dCT images suggested that regions of motion artefact had been sensibly deformed. The two modalities are broadly comparable in terms of the appearance of artefacts and the number of breathing cycles they represent (a single cycle compared with several) so we do not expect registration inaccuracies to arise to the extent they would affect our analysis.

The greatest difficulty in implementing this method comes in defining CTV and OAR structures on the dCT images that are appropriate for dose evaluation purposes. Although the geometric analysis demonstrated that the DIR was able to successfully deform structures, clear differences were identified between the deformed pCT structures and those that were directly outlined on the rCT. There are several reasons for this. Firstly, the rCT CTV volumes did not always include the same anatomy as the original pCT volumes—following partial treatment, the clinician may decide to treat different regions of healthy tissue where subclinical disease is thought to be present. As the algorithm operates to map the originally outlined tissues to their new location, the results will not necessarily match the more recently outlined volumes. In cases where the CTV falls within the heart or lung tissue, this also has an effect on those OAR volumes.

In order to obtain meaningful information on target coverage from the dCT dose distribution, we recommend that the CTV should be outlined by the clinician directly, with due consideration for current clinical objectives (which may differ from those that applied at the planning stage). Information from available structures and imaging may be used to inform this outlining—deformed CTV structures can provide useful information if the GTV and surrounding anatomy has moved or grown, and the original CTV can provide information on where the original CTV anatomy was located. We stress, however, that these structures themselves should not be used as the basis for DVH information.

Secondly, outlining uncertainties in homogenous regions (e.g. regions of atelectasis) where anatomy is not easily distinguishable resulted, in some cases, in large differences for CTV, lung and heart volumes. The issue of inter and intra-operator outlining errors has been highlighted in many studies (Senan *et al* 1999, Bowden *et al* 2002, Giraud *et al* 2002, Steenbakkers *et al* 2006) and the effects were unavoidably present in the comparison between deformed pCT and directly outlined rCT structures.

In addition to this, the registration of homogenous tissues is a greater challenge for an algorithm that uses a similarity measure based on voxel intensities. It is difficult to verify the accuracy of the registration in such regions as there is no reliable gold standard for comparison. While voxelwise dose differences are unlikely to be large in regions of tissue homogeneity, structure deformations could well be inaccurate and clinicians should be aware of this, just as they would be aware that structures directly outlined on the CBCT in homogenous regions may be incorrect. As with other methods of determining the need for a replan (e.g. subjective assessment or direct dose calculation on the CBCT), our method favours scenarios where relevant structures can be distinguished on the CBCT image. If there is substantial uncertainty as to the location of important structures, the clinician should request a rescan in any case.

Finally, as evidenced by one patient in this study (Patient 5, spinal canal  +  5 mm maximum dose difference 9.3 Gy) it is possible for two structures to be very similar but for the dose statistics to be quite different if the structure is located close to a field edge. In this case the directly outlined rCT volume just fell into a treatment field, whereas the deformed structure did not. However, rather than pointing to a DIR issue specifically, this finding highlights the wider issue of plan robustness. Such an error could equally have arisen from set up or outlining and the purpose of the grown PRV volume is to ensure that, if this happens, the serial structure itself (in this case the spinal canal) still remains within tolerance. Indeed, this is shown to be the case in our clinical analysis for this patient, in which we included maximum dose values for both the spinal canal and spinal canal  +  5 mm (table 4). It can be seen here that the spinal canal remains well below the clinical tolerance of 47 Gy throughout, despite the grown PRV volume showing greater variability due to its location close to the field edge.

It is important to note that the infrequency of the image guidance protocol means it is difficult to ascertain if any identified differences arose as a result of random daily variation or a systematic trend. Indeed, weekly image protocols have been shown to be suboptimal in the determination of appropriate setup corrections for this same reason (Higgins *et al* 2011). Therefore, we recommend the implementation of a more frequent image guidance protocol if the method is to be adopted clinically.

There are certain inherent issues with the use of CBCT to assess the ‘dose of the day’. One of these arises as a result of the limited FOV of the CBCT image, which affects both DIR and direct calculation methods. This has been shown not to be a limitation provided the beam arrangement is such that only regions within the FOV are directly irradiated (as is the case for each of the patients in this sample). However, if the method were to be translated to VMAT plans it is likely that truncated regions at the left or right hand side of the patient would be directly irradiated. Whilst missing data outside the FOV can be estimated from pCT data, as was done in this study, the dosimetric effect of directly irradiating these estimated regions has not yet been tested. Further work should therefore be carried out to test the method using VMAT plans. It is hoped that with future advancements in CBCT technology the FOV will be extended and the problem eradicated. The other issue is specific to the use of DIR algorithms in adaptive radiotherapy. In the human body tissues may appear, disappear or change density over the course of treatment. For the lung in particular it has been found that density changes can arise during treatment as a result of pleural effusion and pneumonia or pneumonitis (Møller *et al* 2014)—these changes in density cannot be properly captured by DIR. There is no simple way of addressing this limitation, although semi-automatic adjustments have been suggested in other studies (Veiga *et al* 2016). In the current study, there were no cases where major lung reinflation or collapse had occurred between images that might require correction, as these were excluded at the outset on the basis that a replan would have been required in any case. However, whilst no manual corrections were deemed necessary, further work is required to investigate the effect of more subtle density changes in the lung on dosimetry.

In order to gain confidence in the method it would be desirable to extend this study to a wider participant group. It is unlikely that the necessary number of suitable patients will become available in a single centre, but this may be feasible by way of a larger, multi-institution study. In the meantime, due to the wide variability in the quality of pCT and CBCT data, we advise centres wanting to adopt an adaptation strategy based on DIR to always have a trained operator evaluating the results before they are used to generate dose information for clinical purposes.

## Conclusions

In this study we have demonstrated and validated a tool that can be used clinically to provide quantitative and objective information to help inform the decision as to whether a replan is necessary. We consider that implementation of our method will lead to more consistent decision making for lung cancer patients than currently occurs based on subjective review of on-treatment images alone, reducing the number who are unnecessarily sent for an additional CT and identifying those where adaptive planning could be expected to improve their outcome. With further validation, this tool has the potential for a wide clinical application in the growing field of treatment adaptation.
